# The Relationship Between Trait Emotional Intelligence, Cognition, and Emotional Awareness: An Interpretative Model

**DOI:** 10.3389/fpsyg.2019.01711

**Published:** 2019-07-30

**Authors:** Sergio Agnoli, Giacomo Mancini, Federica Andrei, Elena Trombini

**Affiliations:** ^1^Marconi Institute for Creativity, Alma Mater Studiorum University of Bologna, Bologna, Italy; ^2^Department of Education, Alma Mater Studiorum University of Bologna, Bologna, Italy; ^3^Department of Psychology, Alma Mater Studiorum University of Bologna, Bologna, Italy

**Keywords:** trait emotional intelligence, emotional awareness, fluid intelligence, childhood, Trait Emotional Intelligence Questionnaire – Child Form, Levels of Emotional Awareness Scale for Children, gender, age

## Abstract

Emotional awareness (EA) has been defined as the cognitive skill devoted to the identification and description of one’s own and others’ emotional experiences, an ability that has usually been conceptualized along with the development of cognitive intelligence. Despite this, EA has also been deemed a central constituent of Emotional Intelligence (EI), a construct that captures individual differences in how we perceive, communicate, regulate, and understand our own emotions, as well as the emotions of others. The overlap between the cognitive and the emotional domain in the definition of the EA construct has created several difficulties in both its understanding and its usage, so much so that several questions regarding its nature and structure remain unanswered. The aim of the present work was to test in a unique model the explanatory role of both trait EI and fluid nonverbal intelligence on EA variability in children, controlling for the effect of age, a variable strictly related to cognitive development, as well as gender, which is highly associated with trait EI during childhood. Four hundred and eighty-eight pupils (258 females and 230 males) ranging from 8 to 12 years old completed the Levels of Emotional Awareness Scale for Children, the Trait Emotional Intelligence Questionnaire – Child Form, and a measure of pure non-verbal reasoning ability, the Raven’s Progressive Matrices. The results of a structural equation model showed a positive explanatory power of both Raven and TEIQue scores on EA, revealing that both cognitive intelligence and trait EI significantly explained EA. The same model also showed an indirect effect of age, *via* intelligence scores, on EA, suggesting that the increase of EA with age could be partially imputed to the development of intelligence. Finally, a relation between gender and TEIQue scores confirmed higher trait EI scores in girls than in boys. The implications emerging from this model are discussed.

## Introduction

Emotional intelligence (EI) has been defined as a wide array of individual differences (Hughes and Evans, 2018) that convey the adaptability characteristic of intelligence and the subjective experiences based on emotions. It can be operationalized according to various theoretical frameworks (see Pertides, 2010; Hughes and Evans, 2018). Among these, the trait model (Petrides et al., 2016) conceptualizes EI as a collection of affect-related personality traits measurable with self-reports (Petrides et al., 2007; Hughes and Evans, 2018). Recently, Petrides et al. (2016) provided a comprehensive overview of the fields of application of trait EI, such as the clinical, health, social, educational, and organizational fields. Although much still needs to be investigated and understood, evidence exists of the importance of trait EI not only among the adult population (Andrei et al., 2014) but also among young people given its potential in several real-life domains for both children (e.g., Mavroveli and Sánchez-Ruiz, 2011; Russo et al., 2012; Mancini, 2018) and adolescents (e.g., Mavroveli et al., 2007; Frederickson et al., 2012; Andrei et al., 2015; Mancini et al., 2017).

Specifically, the investigation of trait EI in children has suggested that a higher trait EI level appears to be an important predictive factor of health-related outcomes, such as improved wellbeing and social interactions during development (Andrei et al., 2014), as well as fewer somatic complaints (e.g., Jellesma et al., 2011). Even if gender differences have been highlighted in trait EI during childhood (Mavroveli et al., 2008; Davis and Humphrey, 2012), with higher trait EI scores in females than in males, research demonstrated an overall positive impact of trait EI on children’s adaptive capacities. A number of studies on the role of trait EI through childhood have been conducted in educational contexts, showing that individual differences in trait EI can be relevant for positive adaptation within the classroom, with particular implications for social-emotional competences and for consequent adaptive behaviors with peers (Frederickson et al., 2012). For instance, Petrides et al. (2004) showed that pupils with high trait EI scores were less likely to be expelled from their schools and had a lower frequency of unauthorized absences. Additional studies revealed that high trait EI scores were positively associated with multiple peer ratings for prosocial behavior (Mavroveli et al., 2009). However, data from self-report surveys revealed that a high trait EI is negatively related to bullying (Mavroveli and Sánchez-Ruiz, 2011), victimization attitude (Kokkinos and Kipritsi, 2012) and behavioral problems in general (Poulou, 2014). Several studies also explored the relationship between individual differences in trait EI and academic outcomes. Although the literature still lacks clear and direct results regarding this relationship (Mavroveli et al., 2009; Hansenne and Legrand, 2012), it seems that the construct of trait EI may serve as a moderator of the relationship between intelligence and scholastic performance (Agnoli et al., 2012).

According to Lane (2000a), the most pivotal aspect of EI is probably related to the awareness of emotional experiences in oneself and others. Even if emotional awareness (EA) is not included in either ability or trait EI theoretical formulation, its relation to both constructs has been supported by past research. For instance, with regard to the ability model, higher levels of EA seem to be related to higher emotion perception ability (Lane et al., 1990).

EA has been conceptualized as the cognitive ability to perceive, describe and differentiate one’s own and others’ emotional experiences (Lane and Schwartz, 1987). This construct comprises meta-knowledge about emotional states and experiences (Boden and Thompson, 2015), and it is characterized by attentional and interpretative processes through which the ongoing monitoring, differentiation and analysis of emotions occur. EA can be particularly important during late childhood, especially during the transition through preadolescence and adolescence, as internalizing symptoms, such as anxiety and depression, are often experienced during this period more intensively, and the ability to regulate emotions is not yet fully established (e.g., Lane and Schwartz, 1987; Rieffe and De Rooij, 2012; Eastabrook et al., 2014). Several questions, however, remain unanswered with regard to the relation between both ability and trait EI and EA, especially during development.

The present work is centered on the exploration of the relationship among EA, trait EI and cognitive intelligence during development, focusing on the specific measurement method used to quantify EA during childhood and preadolescence. Are the emotion-related dispositions and self-perceptions defining trait EI able to explain part of the variability in the measurement of EA during childhood and preadolescence over and above the explanatory power of cognitive abilities? This question was addressed through a specific exploration of EA in children and preadolescents.

### The Levels of Emotional Awareness Model

The Levels of Emotional Awareness (LEA) theoretical model that Lane and Schwartz (1987) proposed describes EA as part of the cognitive development domain, unfolding simultaneously with the development of intelligence. In other words, the LEA model maintains that emotional experiences become more differentiated and integrated with age, with representations of emotional states moving from implicit to explicit forms (Lane, 2000b, 2008; Ciarrochi et al., 2003).

The model that Lane and Schwartz (1987) proposed hypothesizes that the organization of emotional experiences is based on the varying complexity of emotional representations. The structure of EA is based on cognitive schemata that are different among individuals and that strictly depend on past experiences expressed through language. On the basis of this theoretical proposition, one’s own ability to be emotionally aware can be identified and measured through the ability to distinguish among various emotional experiences and the level of complexity in one’s description. In the LEA model, emotional experience undergoes a structural transformation following a hierarchical development that defines five levels of emotional awareness (in progressive order): physical sensations, action tendencies, single emotions, blends of emotion, and blends of blends of emotional experience (Lane et al., 1990). During normal development, a continuous process of differentiation and generalization takes place: affective arousal (implicit and preconscious) is initially experienced as bodily sensations and as action tendencies or global states of positive or negative tension. Then, the representation of affective arousal becomes explicit, consciously perceived, and experienced as distinct states of feeling (conscious awareness of one feeling at a time; a mixture of feelings at a time; and an awareness of various mixtures of feelings in ourselves and in others). From this perspective, individuals may differ in their levels of emotional awareness, and disturbances in somato-psychic development may lead to alterations in emotional awareness (Subic-Wrana et al., 2014). The LEA paradigm specifically defines a developmental trajectory of affective development similar to Piaget’s theory of cognitive-sensory development (Piaget and Inhelder, 1972), moving from implicit to explicit processing.

The current literature seems to support the hypothesized relation between cognitive development, defined in terms of the increase with age of the cognitive abilities constituting intelligence, and EA levels (Veirman et al., 2011). In particular, positive but weak correlations between both general and fluid intelligence and EA showed a positive association between these abilities, as well as a relative independence of the two constructs (Mancini et al., 2013). When they were explored during development, a weak association also emerged between EA and both EI abilities and affect-related personality traits (Veirman et al., 2011). Again, this result seems to demonstrate both a positive association and a relative independence between EA and EI during late childhood and early adolescence. EA ended up being associated with specific aspects of intelligence, such as verbal intelligence (Veirman et al., 2011). This last result in particular seems to suggest that EA is related to the cognitive ability to describe emotions with words. However, we cannot exclude that this association is influenced by the nature of the methods used to measure EA, which are usually based on verbal descriptions (Veirman et al., 2011). Notwithstanding, as previously said, EA emerged as being associated with fluid intelligence as well (Mancini et al., 2013), revealing an association between intelligence and EA which is independent from verbal abilities.

EA is usually assessed through self-report-performance tests (Subic-Wrana et al., 2011). The Levels of Emotional Awareness Scale (LEAS; Lane et al., 1990) in particular is a measure that showed reliability and construct validity (Lane et al., 1996). Lower levels of EA have been linked to patients with somatoform disorders, eating disorders, depressive states, alcohol addiction and functional psychosomatic conditions (Bydlowski et al., 2005; Donges et al., 2005; Bochand and Nandrino, 2010; Pasquier and Pedinielli, 2010), as well as to individuals with impairments in mentalization (Subic-Wrana et al., 2010). The LEAS provides a self-assessment of the level of EA ability. As explained in Ciarrochi et al. (2003), the LEAS is neither a performance-based measure nor a self-report measure of affect-related personality dimensions. Rather, it is intended to assess the extent to which people perceive to be aware of emotions in both themselves and others.

Most research on EA has focused on adult and adolescent samples, whereas very few studies have explored its influence on children. In 2005, Bajgar and colleagues implemented a child-friendly version of the LEAS (LEAS-C). The LEAS and LEAS-C differ in the number and content of the scenarios, but the design and scoring procedure are very similar. The reliability of the LEAS-C resulting from validation studies is acceptable and inter-rater reliability is high (Bajgar et al., 2005; Veirman et al., 2011). Similar results on the validity of the tool were also reported in a preliminary study on the Italian validation of LEAS-C (Marchetti et al., 2010). The LEAS-C seems to represent an important research method for exploring emotional awareness during childhood. However, even if the LEA model that Lane proposed is essentially a developmental model (whose roots are founded in the maturative patterns characterizing EA), evidence regarding this model during development is still scant Research on the use of the LEAS-C during childhood can be defined as still being in an early state. Several questions remain unanswered on the model that Lane proposed and on the measure of EA that the LEAS-C provides during development.

### The Present Study

As previously mentioned, Lane’s theory postulates that the internal world of experience finds its roots in integration among the cognitive schemata used to process emotional information. Under this approach, both cognitive and emotional domains should therefore be involved in the development of EA. This theoretical position is in line with other current approaches to the study of EA, which explain EA as a multisided construct defined by an attentional or cognitive dimension and by an emotional dimension (e.g., Boden and Thompson, 2015). When one measures EA during development, these two components should, thus, be taken into account to capture the multidimensional nature of the construct. One could hypothesize that the EA measure that the LEAS-C provides could be defined both by the child’s cognitive developmental stage and by her/his capacity to self-assess her/his and others’ emotional experiences. Given the self-report nature of the instrument, we could expect LEAS-C scores to be explained in part by emotion-related self-perceptions as measured through trait EI scores. On the other hand, if the EA developmental trajectory is defined by children’s cognitive development, we could expect the measure of EA that LEAS-C provides to be explained by age-related changes in children’s cognitive intelligence as well.

Hence, the present work investigated the relationship between trait EI, fluid nonverbal intelligence, and EA during childhood and preadolescence. Specifically, we examined a model in which trait EI and fluid intelligence were modeled as antecedents of EA. Moreover, individual differences in children’s trait EI were considered, with the hypothesis being that they can explain EA scores’ variability related to emotion-related self-perception. Finally, because differences among boys and girls in trait EI scores emerge during development (Mavroveli et al., 2008), the impact of trait EI on EA scores was controlled for gender.

### Hypotheses

**Figure 1:**
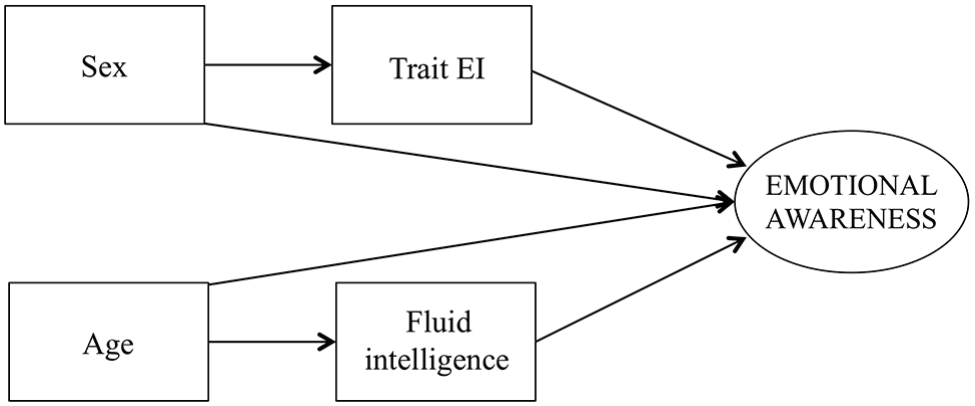
Theoretical model tested in the study.

The main aim of the present study was to test, in a unique model, the explanatory role of trait EI in EA variability during development, controlling for gender differences, and age-related changes in fluid nonverbal intelligence. Our hypotheses are summarized in the theoretical model presented in Figure 1. Specifically, on the basis of past research (Mavroveli et al., 2008; Davis and Humphrey, 2012) we hypothesized that gender differences would be related to differences in trait EI scores and trait EI increase could, in turn, explain an increase in EA scores. In particular, we controlled for the effect of trait EI on EA for gender by exploring both a direct and an indirect effect of gender on EA. Because no gender differences in LEAS-C scores were highlighted in past literature, we did not expect a direct effect of gender on EA. At the same time, we hypothesized that an increase in age would be related to increases in fluid intelligence, which, in turn, could explain EA scores. Besides these independent relationships, we expected that intelligence could mediate the effect of age on EA. This hypothesis is based on the aforementioned LEA model by Lane and Schwartz (1987), which assumes that the enhancement of EA during development is related to the increase of children’s cognitive abilities with age. We specifically tested a mediation effect of age on EA *via* intelligence. Only through an indirect effect of age (*via* intelligence) on EA, could the hypothesis that Lane and Schwartz (1987) proposed be proved. Neither the effect of age on trait EI nor the effect of age on intelligence was tested because, on the basis of past literature, no specific hypothesis could be formulated on these relationships for our tested age. Through the use of a structural equation model approach, our intention was to test whether trait EI scores, controlling for gender, and the development of fluid intelligence can independently explain variability in EA scores during development over and above the role that the other variable exerts.

## Materials and Methods

### Participants

The participants included 514 students ranging in age from 8 to 12 years. All participants were recruited through contacts with primary (3th, 4th, and 5th grade) and middle (6th grade) schools in Northern Italy. Pupils with special educational needs (*n* = 12) and those who were not fluent in Italian (*n* = 14) were excluded from the sample. No further socio-demographic information was collected. Complete data were available for 488 pupils (258 females; mean age = 10.08 years; SD = 1.38 years).

### Measures

#### Levels of Emotional Awareness Scale for Children

Twelve scenarios based on everyday social situations—with each involving two people, oneself and another person—compose the LEAS-C, a tool specifically aimed at children from 8/9 years of age up to preadolescence (Bajgar et al., 2005). An example of a scenario is: “Your teacher tells you that your homework is all wrong and needs to be done again”. Then, two questions are presented: “How would you feel?” and “How would the other person feel?”. Children are asked to respond by indicating how they might feel and how they think the other person might feel in each situation. The scenarios are organized around four emotions (anger, fear, happiness and sadness) presented in mixed order. In particular, in the present study we used the LEAS-C Italian version that Marchetti et al. (2010) developed. Scoring procedures for the LEAS-C are the same as those followed for the adult-based LEAS (Lane et al., 1990). Three scores were assigned to each scenario, reflecting self, others, and overall emotional awareness. Scores for self and others were independently computed on a five-point scale as follows: 0 (no answer and cognitions), 1 (bodily sensations), 2 (actions and general emotional states), 3 (unidimensional emotions), and 4 (blends of emotions). The total score was the largest of the scores for self and others, except in the case of two level-four scores, when a total score of five was given (the maximum possible rating was 60). For the purpose of analyzing the LEAS-C answers, each scenario was coded and scored separately from the remaining scenarios. Four independent, experienced and trained evaluators each assessed about 25% of the responses to the open-ended questions. To aid in the scoring of emotion words, the judges referred to the scoring manual (Bajgar and Lane, 2004) and to its supplement, which includes a glossary of words and specific examples of child response styles. Then, scores awarded were compared. In the case of important discrepancies in codes, coders reviewed the discrepancies, and scores were assigned by consensus. Inter-rater reliability using “Cohen’s к” was comprised between 0.71 and 0.87, which is esteemed as experimentally congruous (Hartmann, 1977). The validity study, gender effects in LEAS-C performance, and relationship between the scale and other emotion assessments are widely reported in Bajgar and Lane (2004). In the current sample, the LEAS-C scales’ internal consistency, calculated using Cronbach’s alpha coefficient, was acceptable: *α* = 0.68 for Total scores, *α* = 0.69 for self-scores, and *α* = 0.73 for others’ scores.

#### Trait Emotional Intelligence Questionnaire – Child Form

Specifically developed for ages 8–12, the Trait Emotional Intelligence Questionnaire – Child Form (TEIQue-CF) is composed of 75 short statements (e.g., “It’s easy for me to show how I feel”) that require a response on a five-point Likert scale, ranging from “completely disagree” to “completely agree” (Mavroveli et al., 2008). The TEIQue-CF showed good levels of internal consistency and temporal stability over a three-month interval (see Mavroveli et al., 2008; Mavroveli and Sánchez-Ruiz, 2011). In the present study the Italian version of the TEIQue-CF was used (Russo et al., 2012). For each participant, a global trait EI score was computed. The reliability of the questionnaire was very high (Cronbach alpha = 0.88). For an introduction to the theory and psychometric properties of the TEIQue as the operationalization vehicle for trait EI, see Petrides (2009) and Petrides and Furnham (2003).

#### Raven’s Colored Progressive Matrices

The CPM is a measure of pure nonverbal reasoning ability that is meant to be independent of the specific cultural or educational context (Raven, 2000). It is widely applied in both practice and the research field due to how easy it is to administer and to interpret in a clear, theoretically relevant way (Raven, 2000). The test is specific for children from about 4/5 to 12 years of age, and it consists of 36 items presented in three sets of 12 each, progressively arranged in order of difficulty to assess the ability to form perceptual relations and reason with analogies. Each item depicts an abstract pattern in a two by two or three-by-three matrix; all cells contain a figure except for the cell in the lower right corner. Participants were asked to find the missing piece in a set of matrices that become progressively more difficult. In accordance with the Italian user manual, CPM has been assessed using answer sheets. A score of 1 was given to each correct answer. A sum of the correct answers has been obtained for every child. Finally, total raw CPM scores were transformed to percentile scores according to age-normative data (see Belacchi et al., 2008). Past research consistently revealed good psychometric properties for this test (Raven, 2000; Belacchi et al., 2008). The CPM demonstrated good validity and reliability indices, and it is considered to be a suitable measure for assessing nonverbal intelligence, particularly that of preschool (Lúcio et al., 2017) and primary school (Cotton et al., 2005) children.

### Design and Procedure

The study received approval from the Ethical Committee of the University of Bologna, and was then presented to school principals and teachers. Informed consent was obtained from parents and caregivers of the schools that decided to participate in the study. For each classroom, a researcher briefly explained answer formats during school hours, and pupils filled out the tests thereafter in the presence of both a researcher and a teacher who was there for safety reasons. The test administration lasted for about 45 min. Pupils were also informed that their participation was voluntary, and that they could decide whether or not to take part in the research. Pupils were additionally assured as to the confidentiality and anonymity of data treatment.

### Data Analysis

Descriptive statistics and Pearson correlations among the study variables were calculated using SPSS 21.0 software (SPSS Inc., Chicago, Illinois, USA). The pattern of relations specified *via* our theoretical model (Figure 1) was investigated through a structural equation model tested with Mplus software (Muthén and Muthén, 1998–2015), with a single observed score for each variable (Trait EI was defined by the TEIQue global score, whereas fluid intelligence was defined by CPM scores) except for the latent variable of Emotional Awareness, which two manifest variables—LEAS self-scores and LEAS scores for others—defined. The main predictors and the hypothesized mediation effects were examined using a bootstrap estimation with 5,000 samples (Muthén and Muthén, 1998–2015). The model fit was estimated with the following indices: the chi-square statistic and the related *p*, the Tucker-Lewis index (TLI), the comparative fit index (CFI), the root mean squared error of approximation (RMSEA), and the standardized root mean squared residual (SRMR). According to Marsh et al. (2004) as well as Hu and Bentler (1999), a good model fit is indicated by a TLI and a CFI above 0.95, an RMSEA below 0.06, an SRMR under 0.08 and a small ratio (< 3) between the chi-square and the degrees of freedom (*χ*^2^/df).

## Results

### Preliminary Analyses

Descriptive statistics and correlations among the study variables are reported in Table 1. Significant correlations between EA for self and EA for others EA emerged, and so did slightly positive associations between the two LEAS-C scales and both cognitive intelligence and trait EI. As expected, a positive association between cognitive intelligence and age emerged, whereas no correlation between cognitive intelligence and trait EI was highlighted.

**Table 1:** Descriptive statistics and correlations among the study variables.

	1	2	3	4	5
1. Self-emotional awareness	–				
2. Others emotional awareness	0.63^**^	–			
3. Cognitive intelligence	0.19^**^	0.15^**^	–		
4. Trait EI	0.12^**^	0.15^*^	0.02	–	
5. Age (months)	0.12^**^	0.06	0.72^**^	−0.01	–
Mean	30.81	29.28	35.89	3.61	126.81
SD	5.14	6.15	9.02	0.39	18.69
Skewness	−0.48	−0.99	0.09	−0.02	0.04
Kurtosis	0.59	1.83	−0.48	−0.15	−1.26

*N = 488*.

* *p < 0.01*;

** *p < 0.001*.

### Structural Equation Model

**Figure 2:**
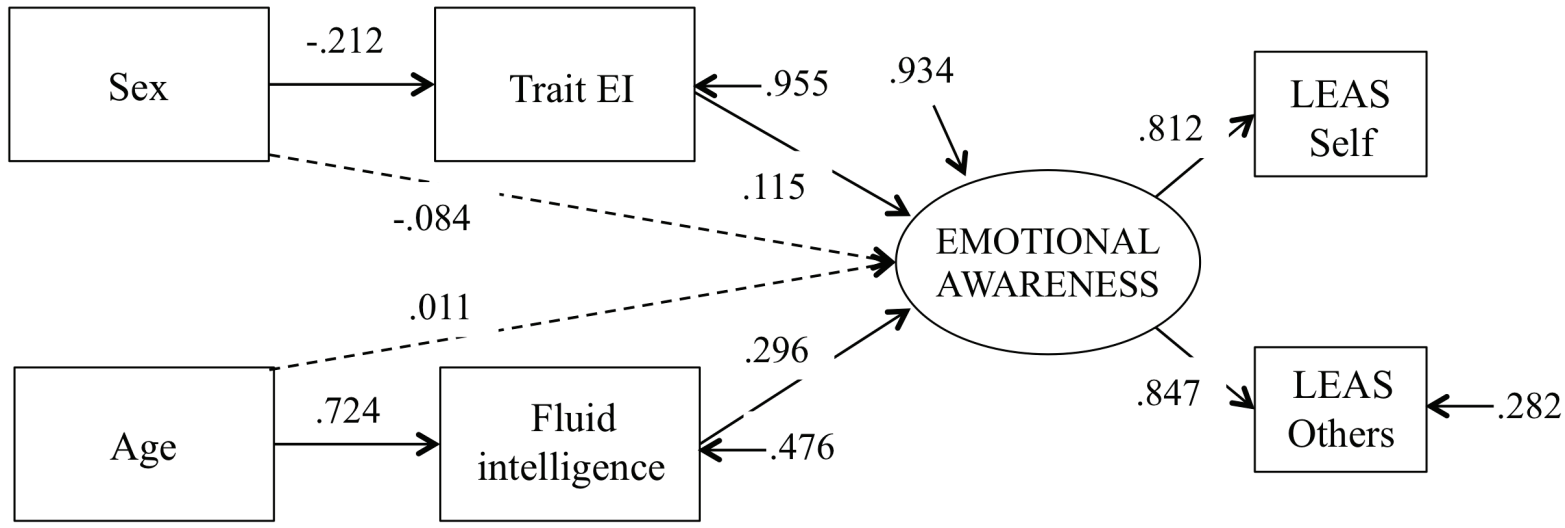
Structural equation model for the prediction of emotional awareness.

The model depicted in Figure 1 was tested. The tested model fit the data well: *χ*^2^(6) = 6.04, *p* = 0.41; TLI = 1.00; *χ*^2^/df = 1.01; CFI = 1.00, RMSEA = 0.01; SRMR = 0.01. All hypotheses were confirmed through the model. As shown in Figure 2, a positive direct link was found between both fluid intelligence (*β* = 0.30, *p* < 0.01) and Trait EI (*β* = 0.11, *p* = 0.03) scores and the latent variable Emotional Awareness, i.e., both cognitive and trait EI emerged as significant predictors of emotional awareness. Moreover, age was positively associated with fluid intelligence scores (*β* = 0.72, *p* < 0.01), displaying an increase of cognitive intelligence with age, whereas the total direct effect of age on Emotional Awareness emerged as not significant (*β* = 0.01, *p* = 0.83). However, a specific indirect effect of age on Emotional Awareness emerged through fluid intelligence scores (*β* = 0.21, *p* < 0.01). It should be noted that, according to Hayes (2009), because no evidence exists that age directly affects Emotional Awareness, we cannot assume that intelligence mediates the effect of age on Emotional Awareness. Instead, we can refer to an indirect effect of age on Emotional Awareness through the effect of intelligence. Moreover sex emerged as being negatively associated with trait EI scores (*β* = −0.21, *p* < 0.01), showing higher EI scores in females than in males. Finally, neither a direct (*β* = −0.08, *p* = 0.11) nor an indirect (*β* = −0.02, *p* = 0.06) effect of sex on Emotional Awareness emerged from the analysis.

## Discussion

The aim of the present work was to consider in a unique model the explanatory role of both trait EI scores, controlling for gender differences and the development of fluid non-verbal intelligence in EA variability in children. The current study’s aim was to explain part of the multifaceted nature of EA and to clarify the measure of EA that the LEAS-C instrument provides during childhood and pre-adolescence. Although past research already demonstrated that EA is subject to developmental changes (Mankus et al., 2016), prior research had not examined whether part of the variability characterizing EA measurement during childhood could be captured through both children’s affect-related personality traits (i.e., trait EI) and cognitive intelligence. This research question lies within the theoretical position explaining EA as a two-sided construct constituted both by an attentional or cognitive dimension, mainly related to skills allowing one to attend to emotions, and by an emotional dimension, mainly related to the ability to understand emotions (Boden and Thompson, 2015). Even if LEA theory mainly posits that EA involves cognitive skills that develop throughout childhood, it also recognizes the close relationship of EA with multiple emotionally adaptive skills.

First of all, the structural equation model tested in the present study showed that both trait EI and cognitive intelligence explain EA. The model was then enriched by a significant indirect effect of age (*via* intelligence) on EA. This result is in line with the LEA theory, which posits that the development of the knowledge or experience of the internal emotional states of self and others is related to the increase with age of the cognitive understanding intended to be a progression toward greater levels of abstraction in thought (Smith et al., 2018). This finding provides the first evidence that the increase of cognitive intelligence could explain a significant part of the variability in EA during childhood and preadolescence. Specifically, it demonstrates that age has an effect on EA only *via* intelligence, suggesting that the increase of EA during development unfolds the development of intelligence as Lane and Schwartz (1987) posited. This result seems to highlight that the increase of EA scores with age is explained by the progressive sophistication of cognitive abilities. It is worth noting that in the present work no effect of age on trait EI was tested, as this affect-related personality dimension has been demonstrated to be highly stable over our tested ages.

Moreover, it is worth highlighting that EA scores as measured through the LEAS-C represent children’s self-perception when it comes to cognitive skills that allow them to be aware of the concept of emotion (Lane and Schwartz, 1987; Lane, 2000a,b; Wright et al., 2018). The present study allowed for disambiguating whether trait EI could explain part of the variability in EA scores during childhood. The results showed that EA scores as measured by the LEAS-C in children and preadolescents are indeed related to participants’ trait EI in that part of their variability is significantly explained by trait EI scores. Moreover, the role of trait EI in explaining EA was independent and incremental to the predictive power of fluid nonverbal intelligence. This effect was controlled in our model for gender, namely for the typical difference characterizing trait EI during childhood, with girls generally showing higher trait EI scores than boys (Mavroveli et al., 2008). Consistent with past research, no direct effect of gender emerged on EA during development. Our results may imply that cognitive skills are not sufficient for understanding and explaining EA and its measure during childhood. In fact, the study showed that trait EI predicts a different source of variability in EA that is independent from cognitive development. Being that trait EI is a personality construct related to the self-perception of affect-related variables (Petrides et al., 2007; Hughes and Evans, 2018), we hypothesized that it can capture a portion of children’s emotional attitudes at the basis of the self-perception of EA skills. As already demonstrated in past research, the broad construct of trait EI led to substantial improvements in the ability to predict behaviors, attitudes and achievement (e.g., Petrides et al., 2007; Agnoli et al., 2015, 2019; Rubaltelli et al., 2015). The results of the present study led to further improvement, as the study showed the capacity of trait EI to predict EA scores in childhood and preadolescence. Thus, it adds to the literature demonstrating the utility of trait EI in explaining EA during development. Moreover, these findings could represent a first step in testing more comprehensive models aimed at understanding the integrative role of cognitive and emotional intelligence on emotional development. For example, starting from these results, it would be particularly intriguing to perform testing with the Integrated Model of Affect-related Individual Differences (IMAID) by Hughes and Evans (2018), which proposes that various EI-related constructs interact with cognitive intelligence to influence affective outcomes, particularly emotion regulation. The exploration of EA during childhood and its association with emotional regulation using integrative frameworks could be a direction for future research, Finally, we auspicate that future research would analyze the development of EA in special samples of participants (e.g., children with learning or cognitive disabilities), which could help to further unravel the weight of trait EI and cognitive skills in the emergence of the awareness of emotional experience in oneself and in others.

A specific research implication for trait EI research is to contribute to greater understanding of the measurement and definition of the trait EI construct in association and in contrast with other emotion-related constructs. Moreover, because of the relevance of trait EI and of EA for children’s well-being, health, and peer relationships, our results may have practical implications particularly relevant for programs and policies addressing the prevention of emotional distress in children and pre-adolescents, by helping to build specific training programs to support emotional competences, acting on multiple layers of children’s emotional experience.

However, we recommend caution in interpreting the results emerging from this work. Our results could indeed be limited by the sample tested in the present study and by the sociocultural context in which the study was conducted. Further studies are required to test and eventually generalize these empirical data in other contexts and countries. In addition, we cannot exclude that the findings of the present study may depend on the chosen instruments and that the generalizability of our results on the explored theoretical constructs could be limited. Again, we hope that further studies will be conducted in order to corroborate our results by exploring the complex relationship among trait EI, cognitive development, and emotional awareness through different sets of instruments.

## Ethics Statement

This study was carried out in accordance with the recommendations of the Italian Psychological Association with written informed consent from the parents of all subjects. The protocol was approved by the ethical committee of the Department of Psychology, University of Bologna, Italy.

## Author Contributions

SA and GM contributed to the design of the work, data collection, drafting the article, and its critical revision. SA contributed to data analysis and interpretation. FA contributed to data collection, drafting the article and its critical revision. ET contributed to final approval of the version to be published.

### Conflict of Interest Statement

The authors declare that the research was conducted in the absence of any commercial or financial relationships that could be construed as a potential conflict of interest.

## References

[ref1] Agnoli S. Franchin L. Rubaltelli E. Corazza G. E. (2019). The emotionally intelligent use of attention and affective arousal under creative frustration and creative success. Personal. Individ. Differ. 142, 242–248. 10.1016/j.paid.2018.04.041

[ref2] Agnoli S. Mancini G. Pozzoli T. Baldaro B. Russo P. M. Surcinelli P. (2012). The interaction between emotional intelligence and cognitive ability in predicting scholastic performance in school-aged children. Personal. Individ. Differ. 53, 660–665. 10.1016/j.paid.2012.05.020

[ref3] Agnoli S. Pittarello A. Hysenbelli D. Rubaltelli E. (2015). “Give, but give until it hurts”: the modulatory role of trait emotional intelligence on the motivation to help. PLoS One 10, 1–22. 10.1371/journal.pone.0130704 PMC4487050 26121350

[ref4] Andrei F. Mancini G. Baldaro B. Trombini E. Agnoli S. (2014). A systematic review on the predictive utility of the trait emotional intelligence questionnaire (TEIQue). Appl. Psychol. Bull. 271, 2–29.

[ref5] Andrei F. Mancini G. Mazzoni E. Russo P. M. Baldaro B. (2015). Social status and its link with personality dimensions, trait emotional intelligence and scholastic achievement in children and early adolescents. Learn. Individ. Differ. 42, 97–105. 10.1016/j.lindif.2015.07.014

[ref6] Bajgar J. Ciarrochi J. Lane R. D. Deane F. (2005). Development of the levels of emotional awareness scale for children (LEAS-C). Br. J. Dev. Psychol. 23, 569–586. 10.1348/026151005X35417, PMID: 21214598

[ref7] Bajgar J. Lane R. (2004). The levels of emotional awareness scale for children (LEAS-C): Scoring manual supplement. Wollongong, Australia: University of Wollongong, Illawarra Institute for Mental Health.

[ref500] Belacchi C. Scalisi T. G. Cannoni E. Cornoldi C. (2008). Manuale CPM. Coloured progressive matrices. Standardizzazione italiana. PMID: 21214598

[ref8] Bochand L. Nandrino J. L. (2010). Levels of emotional awareness in alcohol-dependent patients and abstinent alcoholics. L’Encephale 36, 334–339. 10.1016/j.encep.2009.12.013, PMID: 20850605

[ref9] Boden M. T. Thompson R. J. (2015). Facets of emotional awareness and associations with emotion regulation and depression. Emotion 15, 399–410. 10.1037/emo0000057, PMID: 25706832

[ref10] Bydlowski S. Corcos M. Jeammet P. Paterniti S. Berthoz S. Laurier C.. (2005). Emotion-processing deficits in eating disorders. Int. J. Eat. Disord. 37, 321–329. 10.1002/eat.20132, PMID: 15856501

[ref11] Ciarrochi J. Caputi P. Mayer J. D. (2003). The distinctiveness and utility of a measure of trait emotional awareness. Personal. Individ. Differ. 34, 1477–1490. 10.1016/S0191-8869(02)00129-0

[ref12] Cotton S. M. Kiely P. M. Crewther D. P. Thomson B. Laycock R. Crewther S. G. (2005). A normative and reliability study for the Raven’s coloured progressive matrices for primary school aged children from Victoria, Australia. Personal. Individ. Differ. 39, 647–659. 10.1016/j.paid.2005.02.015

[ref501] Davis S. K. Humphrey N. (2012). The influence of emotional intelligence (EI) on coping and mental health in adolescence: Divergent roles for trait and ability EI. J. Adolesc. 35, 1369–1379. PMID: 22704492 10.1016/j.adolescence.2012.05.007

[ref13] Donges U. S. Kersting A. Dannlowski U. Lalee-Mentzel J. Arolt V. Suslow T. (2005). Reduced awareness of others’ emotions in unipolar depressed patients. J. Nerv. Ment. Dis. 193, 331–337. 10.1097/01.nmd.0000161683.02482.19, PMID: 15870617

[ref14] Eastabrook J. M. Flynn J. J. Hollenstein T. (2014). Internalizing symptoms in female adolescents: associations with emotional awareness and emotion regulation. J. Child Fam. Stud. 23, 487–496. 10.1007/s10826-012-9705-y

[ref15] Frederickson N. Petrides K. V. Simmonds E. (2012). Trait emotional intelligence as a predictor of socioemotional outcomes in early adolescence. Personal. Individ. Differ. 52, 323–328. 10.1016/j.paid.2011.10.034

[ref16] Hansenne M. Legrand J. (2012). Creativity, emotional intelligence, and school performance in children. Int. J. Educ. Res. 53, 264–268. 10.1016/j.ijer.2012.03.015

[ref17] Hartmann D. P. (1977). Considerations in the choice of inter-observer reliability estimates. J. Appl. Behav. Anal. 10, 103–116. 10.1901/jaba.1977.10-103, PMID: 16795538 PMC1311156

[ref503] Hayes A. F. (2009). Beyond Baron and Kenny: Statistical mediation analysis in the new millennium. Commun. Monogr. 76, 408–420. 10.1080/03637750903310360, PMID: 16795538

[ref18] Hu L. T. Bentler P. M. (1999). Cutoff criteria for fit indexes in covariance structure analysis: conventional criteria versus new alternatives. Struct. Equ. Model. Multidiscip. J. 6, 1–55. 10.1080/10705519909540118

[ref19] Hughes D. J. Evans T. R. (2018). Putting ‘emotional intelligences’ in their place: introducing the integrated model of affect-related individual differences. Front. Psychol. 9:2155. 10.3389/fpsyg.2018.02155, PMID: 30487765 PMC6246631

[ref20] Jellesma F. C. Rieffe C. Meerum Terwogt M. Westenberg P. M. (2011). Children’s sense of coherence and trait emotional intelligence: a longitudinal study exploring the development of somatic complaints. Psychol. Health 26, 307–320. 10.1080/08870440903411021, PMID: 20309781

[ref22] Kokkinos C. M. Kipritsi E. (2012). The relationship between bullying, victimization, trait emotional intelligence, self-efficacy and empathy among preadolescents. Soc. Psychol. Educ. 15, 41–58. 10.1007/s11218-011-9168-9

[ref23] Lane R. D. (2000a). “Levels of emotional awareness: neurological, psychological, and social perspectives” in The handbook of emotional intelligence: Theory, development, assessment and application at home, school, and in the workplace. eds. Bar-On R. Parker J. D. A. (San Francisco: Jossey-Bass), 171–191.

[ref24] Lane R. D. (2000b). “Neural correlates of conscious emotional experience” in Cognitive neuroscience of emotion. eds. Lane R. D. Nadel L. (New York: Oxford University Press), 345–370.

[ref25] Lane R. D. (2008). Neural substrates of implicit and explicit emotional processes: a unifying framework for psychosomatic medicine. Psychosom. Med. 70, 214–231. 10.1097/PSY.0b013e3181647e44, PMID: 18256335

[ref26] Lane R. D. Quinlan D. M. Schwartz G. E. Walker P. A. (1990). The levels of emotional awareness scale: a cognitive-developmental measure of emotion. J. Pers. Assess. 55, 124–134. 10.1080/00223891.1990.9674052, PMID: 2231235

[ref27] Lane R. D. Schwartz G. (1987). Levels of emotional awareness: a cognitive-developmental theory and its application to psychopathology. Am. J. Psychiatr. 144, 133–143. 10.1176/ajp.144.2.133, PMID: 3812780

[ref28] Lane R. D. Sechrest L. Reidel R. Weldon V. Kaszniak A. Schwartz G. E. (1996). Impaired verbal and nonverbal emotion recognition in alexithymia. Psychosom. Med. 58, 203–210. 10.1097/00006842-199605000-00002, PMID: 8771618

[ref29] Lúcio P. S. Cogo-Moreira H. Puglisi M. Polanczyk G. V. Little T. D. (2017). Psychometric investigation of the Raven’s colored progressive matrices test in a sample of preschool children. Assessment 1–11. 10.1177/1073191117740205 29121785

[ref30] Mancini G. (2018). Trait emotional intelligence and draw-a-person emotional indicators: a first study on 8-year-old Italian children. Child Indic. Res. 2018, 1–13. 10.1007/s12187-018-9601-0

[ref31] Mancini G. Agnoli S. Trombini E. Baldaro B. Surcinelli P. (2013). Predictors of emotional awareness during childhood. Health 5, 375–380. 10.4236/health.2013.53050

[ref32] Mancini G. Andrei F. Mazzoni E. Biolcati R. Baldaro B. Trombini E. (2017). Brief report: trait emotional intelligence, peer nominations, and scholastic achievement in adolescence. J. Adolesc. 59, 129–133. 10.1016/j.adolescence.2017.05.020, PMID: 28618332

[ref33] Mankus A. M. Boden M. T. Thompson R. J. (2016). Sources of variation in emotional awareness: age, gender, and socioeconomic status. Personal. Individ. Differ. 89, 28–33. 10.1016/j.paid.2015.09.043, PMID: 26500384 PMC4612349

[ref34] Marchetti A. Valle A. Massaro D. Castelli I. (2010). Emotional awareness in school-aged children: a contribution to the Italian validation of the LEAS-C. Ric. Psicol. 4, 555–574. 10.3280/RIP2010-004005

[ref35] Marsh H. W. Hau K. T. Wen Z. (2004). In search of golden rules: comment on hypothesis-testing approaches to setting cutoff values for fit indexes and dangers in overgeneralizing Hu and Bentler’s (1999) findings. Struct. Equ. Model. 11, 320–341. 10.1207/s15328007sem1103_2

[ref36] Mavroveli S. Petrides K. V. Rieffe C. Bakker F. (2007). Trait emotional intelligence, psychological well-being and peer-rated social competence in adolescence. Br. J. Dev. Psychol. 25, 263–275. 10.1348/026151006X118577

[ref37] Mavroveli S. Petrides K. V. Sangareau Y. Furnham A. (2009). Exploring the relationships between trait emotional intelligence and objective socio-emotional outcomes in childhood. Br. J. Educ. Psychol. 79, 259–272. 10.1348/000709908X368848, PMID: 18950549

[ref38] Mavroveli S. Petrides K. V. Shove C. Whitehead A. (2008). Validation of the construct of trait emotional intelligence in children. Eur. Child Adolesc. Psychiatry 17, 516–526. 10.1007/s00787-008-0696-6, PMID: 18563477

[ref39] Mavroveli S. Sánchez-Ruiz M. J. (2011). Trait emotional intelligence influences on academic achievement and school behaviour. Br. J. Educ. Psychol. 81, 112–134. 10.1348/2044-8279.002009, PMID: 21199490

[ref40] Muthén L. K. Muthén B. O. (1998–2015). Mplus user’s guide. 7th Edn. Los Angeles, CA: Muthén & Muthén.

[ref41] Pasquier A. Pedinielli J. L. (2010). Exploratory study of relations between emotional awareness, social sharing of emotions, anxious and depression states. L’Encephale 36, D97–D104. 10.1016/j.encep.2009.01.007, PMID: 20513467

[ref42] Pertides K. V. (2010). Trait emotional intelligence theory. Ind. Organ. Psychol. 3, 136–139. 10.1111/j.1754-9434.2010.01213.x

[ref43] Petrides K. V. (2009). “Psychometric properties of the trait emotional intelligence questionnaire” in Advances in the assessment of emotional intelligence. eds. Stough C. Saklofske D. H. Parker J. D. (New York: Springer).

[ref44] Petrides K. V. Frederickson N. Furnham A. (2004). The role of trait emotional intelligence in academic performance and deviant behavior at school. Personal. Individ. Differ. 36, 277–293. 10.1016/S0191-8869(03)00084-9

[ref45] Petrides K. V. Furnham A. (2003). Trait emotional intelligence: behavioural validation in two studies of emotion recognition and reactivity to mood induction. Eur. J. Personal. 17, 39–57. 10.1002/per.466

[ref46] Petrides K. V. Mikolajczak M. Mavroveli S. Sanchez-Ruiz M. J. Furnham A. Pérez-González J. C. (2016). Developments in trait emotional intelligence research. Emot. Rev. 8, 335–341. 10.1177/1754073916650493

[ref47] Petrides K. V. Pita R. Kokkinaki F. (2007). The location of trait emotional intelligence in personality factor space. Br. J. Psychol. 98, 273–289. 10.1348/000712606X120618, PMID: 17456273

[ref48] Piaget J. Inhelder B. (1972). Die Psychologie des Kindes. Olten: Walter Publishers.

[ref49] Poulou M. S. (2014). How are trait emotional intelligence and social skills related to emotional and behavioural difficulties in adolescents? Educ. Psychol. 34, 354–366. 10.1080/01443410.2013.785062

[ref50] Raven J. (2000). The Raven’s progressive matrices: change and stability over culture and time. Cogn. Psychol. 41, 1–48. 10.1006/cogp.1999.0735, PMID: 10945921

[ref51] Rieffe C. De Rooij M. (2012). The longitudinal relationship between emotion awareness and internalising symptoms during late childhood. Eur. Child Adolesc. Psychiatry 21, 349–356. 10.1007/s00787-012-0267-8, PMID: 22466448 PMC3370159

[ref504] Rubaltelli E. Agnoli S. Franchin L. (2015). Sensitivity to affective information and investors’ evaluation of past performance: An eye-tracking study. J. Behav. Decis. Making 29, 295–306. 10.1002/bdm.1885, PMID: 17456273

[ref52] Russo P. M. Mancini G. Trombini E. Baldaro B. Mavroveli S. Petrides K. V. (2012). Trait emotional intelligence and the big five: a study on Italian children and preadolescents. J. Psychoeduc. Assess. 30, 274–283. 10.1177/0734282911426412

[ref53] Smith R. Quinlan D. Schwartz G. E. Sanova A. Alkozei A. Lane R. D. (2018). Developmental contributions to emotional awareness. J. Pers. Assess. 101, 1–9. 10.1080/00223891.2017.1411917 29388809

[ref54] Subic-Wrana C. Beutel M. E. Brähler E. Stöbel-Richter Y. Knebel A. Lane R. D.. (2014). How is emotional awareness related to emotion regulation strategies and self-reported negative affect in the general population? PLoS One 9:e91846. 10.1371/journal.pone.0091846, PMID: 24637792 PMC3956759

[ref55] Subic-Wrana C. Beutel M. E. Garfield D. A. Lane R. D. (2011). Levels of emotional awareness: a model for conceptualizing and measuring emotion-centered structural change. Int. J. Psychoanal. 92, 289–310. 10.1111/j.1745-8315.2011.00392.x 21518361

[ref56] Subic-Wrana C. Beutel M. E. Knebel A. Lane R. D. (2010). Theory of mind and emotional awareness deficits in patients with somatoform disorders. Psychosom. Med. 72, 404–411. 10.1097/PSY.0b013e3181d35e83, PMID: 20223925

[ref57] Veirman E. Brouwers S. A. Fontaine J. R. J. (2011). The assessment of emotional awareness in children. Validation of the levels of emotional awareness scale for children. Eur. J. Psychol. Assess. 27, 265–273. 10.1027/1015-5759/a000073

[ref502] Wright R. Riedel R. Sechrest L. Lane R. D. Smith R. (2018). Sex differences in emotion recognition ability: The mediating role of trait emotional awareness. Motiv. Emot. 42, 149–160.

